# Hemostatic profile and thromboembolic risk in healthy dogs treated with prednisone: a randomized controlled trial

**DOI:** 10.1186/1746-6148-9-268

**Published:** 2013-12-31

**Authors:** Felipe Gazza Romão, Elza Fernanda Campos, Claudio Roberto Scabelo Mattoso, Regina Kiomi Takahira

**Affiliations:** 1 Department of Veterinary Clinics, Botucatu Veterinary Medicine School, University Estadual Paulista – UNESP, Botucatu, Brazil; 2 Departament of Veterinary Medicine, Lages Veterinary Medicine School, University do Estado de Santa Catarina – UDESC, Lages, Brazil

**Keywords:** Platelets, Hemostasis, Prednisone, Thromboembolism

## Abstract

**Background:**

Thrombosis has been associated to some diseases like hyperadrenocorticism (HAC). Several drugs can alter the balance, such as the corticosteroid prednisone, used mainly for its anti-inflammatory and immunosuppressive effects. It is known that hypercortisolism can stimulate thrombi formation by increasing coagulation factors and decreasing fibrinolysis. However it is not known how prednisone administration affects hemostasis in dogs and if it is dose dependent. The aim of this study, therefore, was to demonstrate the effects of prednisone administration on dogs’ hemostatic profile.

**Results:**

Significant decrease of antithrombin levels was observed in both groups (anti-inflammatory and immunosuppressive doses) after 15 days of treatment. An increase of platelet aggregation was observed in dogs receiving immunosuppressive doses of prednisone (Group II).

**Conclusions:**

From the results obtained in our study, it is not possible to infer that hypercortisolism can increase the thromboembolic risk, despite the decreased anticoagulant factors (antithrombin levels).

## Background

Despite being more common in humans, thromboembolic risk has been more associated to hypercortisolism in dogs than in humans [1]. Prospective studies concerning overall effects of corticosteroids on hemostasis are rare in dogs. In human, this risk has been associated to an increase of factors V, VIII, IX, XI, XII, and prothrombin, von Willebrand factor, and platelet hyperactivity [2]. Most studies do not clearly define if hemostatic changes in animals presenting Cushing’s syndrome are associated to direct effects of corticosteroids or secondarily to endogenous elevation observed in cases of HAC, including those caused by metabolic imbalances or mineralocorticoid action.

Prednisone is a corticoid widely used in clinics and veterinary hospital settings. In spite of this fact, only a few studies have compared the effects of long-term exogenous administration of prednisone (15 days protocols) at anti-inflammatory and immunosuppressive doses.

Therefore, the aim of this study was to assess the effects of prednisone on platelet count and function, coagulation profile and the fibrinolytic system (Table 1) in healthy dogs, in order to determine the thromboembolic risk associated to corticotherapy.

**Table 1:** Medians, p-values and interquartile range (p25; p75) values of hemostatic profile on pre (M0) and post-treatment (M1) moments of groups I and II

	**M0**	**M1**	**M0**	**M1**
Platelet aggregation (%)	73.00 (71.25;81.5)	81.00 (p = 0.41) (78.03;86)	64.50 (61;86.75)	83.00* (p = 0.03) (77.5;92.25)
Antithrombin (%)	92.3 (87.32;95.05)	76.25* (p = 0.02) (71.48;82.18)	125.25 (106.15;132.15)	77.3* (p = 0.005) (71.67;82.07)
Cortisol (μg/dL)	1.19 (0.97;1.31)	3.28* (p = 0.003) (2.65;7.46)	1.97 (1.38;3.21)	14.97* (p = 0.003) (5.66;21.59)
Factor VIII (%)	110.33 (93.19;171.57)	88.38 (p = 0.36) (60.55;93.19)	161.35 (90.89;243.80)	151.96 (p = 0.77) (135.09;160.36)
Fibrinogen (mg/dL)	234.52 (186.14;234.52)	298.08 (p = 0.30) (158.19;328.21)	234.52 (183.53;348.66)	184.09 (p = 0.09) (162.19;205.77)
vWF (%)	187.58 (148.76;241.42)	111.14* (p = 0.049) (82.9;142.46)	112.21 (100.19;133.48)	79.88 (p = 0.14) (59.09;136.26)
FDP (mg/dL)	0 (0;1)	0 (p = 0.62) (0;2)	1.50 (0.25;2)	1.50 (p = 1.00) (1;2)
Platelets (/μL)	228.000 (204.000;292.500)	226.500 (p = 0.56) (195.250;343.250)	294.500 (217.031;421.000)	348.000 (p = 0.84) (261.750;387.387)
PT (s)	11.80 (11.03;13.05)	10.50 (p = 0.15) (9.7;11.2)	9.90 (9;10.4)	8.50 (p = 0.09) (7.5;9.95)
OMBT (s)	74.50 (59.5;88.75)	86.00 (p = 0.22) (62.25;107.5)	80.00 (55;84.25)	75.50 (p = 0.62) (66.25;100)
TT (s)	14.75 (12.25;17.25)	10.38 (p = 0.07) (9.70;11.40)	11.30 (9.7;12.5)	9.65 (p = 0.18) (9.08;9.98)
aPTT (s)	18.00 (15.75;19.21)	16.00 (p = 0.62) (15.23;18)	14.00 (13.8;14.9)	14.70 (p = 0.64) (13.4;16.23)

vWF: von Willebrand Factor; FDP: Fibrin degradation products; PT: Prothrombin time; OMBT: Oral mucosa bleeding time; TT: Thrombin time; aPTT: Activated Partial Thromboplastin time. *Statistical difference (p < 0,05).

## Methods

### Animals

For this study, twenty healthy mongrel neutered dogs, both sexes (15 females, 5 males), between one and eight years-old, mean age five years-old, from the university kennel and from volunteer owners, were used. The normal health status was established based on the results of physical examination, complete blood count, and biochemical profile (urea, creatinine, ALT, ALP, GGT, glucose, and albumin). Bitches in estrous, proestrous, pregnant, lactating, and also obese animals were excluded from the study. Animals were divided into two groups: Group I, composed of 10 animals, receiving prednisone at anti-inflammatory dose (1.0 mg/kg/BID PO), for 15 days; Group II, with 10 animals, receiving prednisone at immunosuppressive dose (2.0 mg/kg/BID PO), for 15 days. Both groups were evaluated for blood count, serum biochemistry tests, hemostatic profile, and urinalysis. These tests were carried out at day 0 (moment 0) and after treatment completion (day 15 – moment 1). All performed procedures were previously approved by the Ethics Committee on Animal Use of the School of Veterinary Medicine and Animal Science, Univ. Estadual Paulista.

### Blood collection

After tricotomy and local antisepsis, blood samples from the jugular vein were directly collected into vacuum tubes containing one of the following: EDTA for platelet and complete blood count; sodium citrate 3.2% in order to obtain platelet-rich plasma (PRP) for platelet aggregation test and platelet-poor plasma for the determination of fibrinogen levels, factor VIII activity, vWF, AT, and FDPs; no anticoagulant in order to measure cortisol levels. All blood samples were collected between 8 and 10 AM. At day 15, blood sample collection coincided with a 2-hour period after prednisone administration. Platelet-rich plasma was obtained from samples stored at room temperature and processed up to two hours after blood sample collection; citrated platelet-poor plasma was obtained from blood samples kept in ice bath and separated by centrifugation up to one hour after blood sample collection. Samples were immediately frozen in liquid nitrogen and stored in a freezer at -80°C until processing.

### Complete blood count (CBC)

The complete blood count was carried out on an automatic cell counter (model Hemascreen, Ebram Laboratory Products^®^), and included red cell count, hemoglobin, MCV, MCHC, and RDW, total white cell blood count and platelet count. Platelet count was estimated during blood smear evaluation to ensure accuracy of the automated platelet count. The hematocrit was measured by the microhematocrit method and plasma protein levels were determined by refractometry. Differential count was carried out counting 100 cells on a stained blood smear (Panótico rápido – Laborclin^®^).

### Platelet aggregation

Platelet aggregation was evaluated by the addition of 50 μL of ADP (ADP Reagent – Helena Laboratories^®^) to 400 μL of PRP (standardized for a 100.000 to 300.000 platelets/μL count). Platelet response to the agonist was measured by turbidimetric aggregometry (Netlab 2000 - ZÊNITE^®^) and expressed as aggregation percentage.

### Oral mucosa bleeding time (OMBT)

The OMBT was evaluated according to Mattoso [3]. Dogs were restrained on sternal or lateral recumbence, the upper lip was then everted and a standard incision was made vertically (perpendicular to lip border), directly under the upper fang, using a lancet (Triplett^®^). The chronometer was started immediately after the incision and blood was absorbed in a circular filter paper located 1 to 2 millimeters away from the incision. The chronometer was stopped once the bleeding stopped.

### Hemostatic profile

The hemostatic profile included determination of factor VIII activity, fibrinogen levels, PT, aPTT, TT (Helena Laboratories^®^), and AT (Siemens^®^) using commercial kits, according to recommendations provided by the manufacturers. The FDPs were evaluated by latex agglutination test, according to recommendations provided by the manufacturer (Diagnostica Stago^®^).

### Von Willebrand’s Factor (vWF)

The vWF antigen was measured by direct ELISA method (ELISA Multiskan EX Original - Labsystems^®^) using canine anti-vWF antibody (Sheep anti-canine VWF-Research Diagnostics Inc^®^) onto specific plates (Nunc^®^ - Immuno Plates), according to the technique recommended by the manufacturer. Control samples were kindly supplied by Dr. James Catalfamo.

### Serum cortisol levels

Serum cortisol levels were measured by radioimmunoassay method, using commercial kits for solid-phase cortisol (Coat – A Count, Siemens^®^), in order to guarantee that significant blood levels of prednisone were successfully reached.

### Statistical analysis

Animals were randomly assigned to one of the two groups and the results were submitted to the descriptive analysis laboratory (median and interquartile range). Due to the presence of variable degrees of asymmetry, the Wilcoxon test was used to compare the median of each response variables between study groups moments 0 and 1. For each study group, the Wilcoxon signed rank test for paired samples was used to make comparisons between moments [4]. The statistical analysis was made with PROC NPAR1WAY and PROC UNIVARIATE (SAS Institute^®^) procedures.

## Results and discussion

Antithrombin (AT) is the major plasma inhibitor among serine proteases and was the only hemostatic value that were significantly different in both groups. Its measurement represents the only available test able to assess the inhibitory factors of coagulation. Healthy dogs present values greater than 80%; values below 50% are associated to a greater thromboembolic risk [5]. Antithrombin levels are significantly decreased after prednisone administration, which is in accordance to the findings from another study that found decreased antithrombin levels in animals with hyperadrenocorticism compared to healthy dogs [6]. This decrease could have been caused by an impairment on hepatic protein synthesis [7]. The median was less than 80% in both groups and none of the animals showed antithrombin activity below 50%. On the other hand, Klose et al. did not show a significant statistical difference comparing healthy dogs, patients with hyperadrenocorticism, and sick animals receiving treatment [2].

Although many studies correlate hypercortisolism to thrombocytosis [8,9], our study did not show a significant increase of platelet count after receiving prednisone treatment, similar to Klose et al. who studied dogs with hyperadrenocorticism [2]. The correlation between increased platelet count and hypercortisolism is extremely controversial, as most of the studies aimed to demonstrate this correlation have evaluated patients with immune-mediated disorders (e.g., immune-mediated thrombocytopenia), and consequently, an increase of platelet number would be expected after corticotherapy due to a diminution of platelet destruction [10].

The present study did not evidence any significant difference for OMBT after prednisone therapy in both groups. This data corroborates the findings of Mackin et al. who studied healthy dogs, and Eberle and Mischke, who measured the capillary bleeding time in the toe of dogs with lymphoma, since they did not find any significant change on bleeding time when prednisone was used as part of the chemotherapeutic protocol [11,12]. It is important to note that OMBT is not a sensitive test to detect minor changes on platelet function.

In the present study, a significant decrease of von Willebrand factor (vWF) levels was observed after prednisone administration only on Group I (anti-inflammatory dose). These findings corroborate those from Giordano et al. [13], who observed a decrease of vWF antigen levels after multiple chemotherapy agents, including prednisone, in children with acute lymphocytic leukemia. Nevertheless, these changes could have been caused by other drugs; in addition, vWF values observed in our study were within reference range for dogs [14].

It is important to note that the vast majority of the studies that found increased vWF after corticotherapy, studied patients who had inflammatory disorders with concomitant endothelial injury that could explain such increased values. This can be inferred by the correlation between vWF levels and C-reactive protein in these patients; therefore, the decrease of vWF levels could be explained by prednisone anti-inflammatory effects. It is not possible to infer why we did not find any significant decrease associated to immunosupressive dose; moreover, Jilma et al. observed an important increase of vWF after the administration of dexamethasone at high-doses to healthy men [15], although another study has demonstrated that the increase of VWF levels is not a constant feature of Cushing syndrome, since some patients may have normal VWF levels in spite of high circulating cortisol levels. This fact may be explained because the normal range of VWF levels is extremely wide since VWF concentrations may be modulated by several genetic and environmental factors [16]. Moreover, dexamethasone has a stronger glucocorticoid effect than prednisone, which is why these aspects may explain a more protective role of dexamethasone concerning the development of thrombotic events [7].

A significant increase of platelet aggregation was observed only in the group treated with the immunosuppressive dose of prednisone. Thong et al. did not find any significant change on platelet aggregation in patients receiving prednisone for two days [17], while Shapiro et al. did not reveal any alteration on platelet aggregation in patients treated with a chemotherapeutic protocol containing prednisone [18]. However, it is known that this protocol include vincristine, which inhibits platelet aggregation [19], suggesting an antagonistic effect between those two drugs that could have prevented the prothrombotic effect of prednisone. Another study evidenced an inhibition of platelet aggregation in rats receiving variable doses of dexamethasone for five days [20].

The agonist-induced platelet aggregation, as adenosine diphosphate (ADP), is an important *in vivo* indicator of platelet function. The turbidimetric method used in the present study has greater clinical relevance and it is easier to standardize than the electrical impedance method. Other agonists can be used, such as ristocetin and epinephrine, but dogs platelets are much less responsive to these substances than human platelets; therefore, ADP is the first choice agonist for platelet aggregation test in dogs [21].

The activated partial thromboplastin time (aPTT) did not reveal any statistically significant change in both groups. This fact confirms the findings of another study that compared this parameter in healthy patients and individuals with hyperadrenocorticism under treatment [2]; however it contradicts data from Manetti et al. and van der Pas et al. both showing shorter aPTT in patients with hypercortisolism [22,23]. This can be explained by increased factor VIII activity and fibrinogen levels, which are important components of the coagulation cascade [24]. In dogs, aPTT is usually more sensitive to deficiencies of factors VIII and IX, and it can be prolonged when there is an activity range of 60 to 75% of these factors [25]. It is not possible to state that an increase on these factor activities could lead to a shortened aPTT, since this test was designed to identify deficiencies on components of this coagulation pathway.

Prednisone therapy has been related to increased factor VIII activity in many studies [8,9,22,23,26-28], a fact that seems to be correlated to cortisol levels, returning to normal levels after treatment completion [6]. A decreased factor VIII activity was noted in both groups, although no significant difference was observed, confirming the data reported by Jacoby et al. in dogs, and in humans by other authors [6,29].

The present study did not evidence any significant difference (p < 0,05) on PT values after treatment completion in both groups, confirming the data reported by Klose et al. [2], which could be explained by the fact that this test is closely related to fibrinogen levels and factor VII, V, and X activities (common and intrinsic pathways of coagulation) [24]. However, fibrinogen levels were not significantly different in both groups after administration of prednisone. The other coagulation factors cited were not evaluated in our study, preventing a complete relation between PT and these factors.

The thrombin time (TT) assesses the common pathway of coagulation; therefore, it is an indirect test of plasma fibrinogen levels [24]. It is not widely used in studies involving hypercoagulability and administration of corticosteroids, probably because most of these studies preferred to evaluate fibrinogen levels in order to assess this pathway. There was no statistical difference in TT after treatment completion in both groups.

Fibrinogen levels were not significantly different after treatment completion when compared to the control moment in both groups. This fact supports the results obtained by Klose et al. [2]. Other authors confirmed an increase of fibrinogen levels, but most of them evaluated the hemostatic profile on patients with hyperadrenocorticism [6,22,23,26,30]; nevertheless, Giordano et al. found a significant decrease of plasma fibrinogen levels [13] confirming the findings of other study [31]. These studies suggest liver dysfunction caused by prednisone administration, leading to a decrease of hepatic fibrinogen synthesis.

There were no significant changes regarding FDPs values between different moments. This data confirms the findings of a study that evaluated patients treated with a chemotherapeutic protocol containing prednisone [27]. The hypercoagulable condition evidenced by decreased levels of antithrombin does not seem to have led to a thrombotic event.

The hypercoagulability should not be explained only by the increased procoagulant factors, but also by the inhibition of the fibrinolytic system and decreased antithrombin activity [6]. It is extremely difficult to correlate hypercortisolism to thromboembolism in dogs, since the studies that evaluated these effects are vastly based on data from human studies. There are other factors involved in the thromboembolic risk than just increased procoagulant factors and decreased fibrinolytic factors, such as systemic arterial hypertension, a more prominent sign in human beings with hyperadrenocorticism than in dogs suffering from the same condition. The thromboelastography (TEG) is a reliable method to define the increase of thromboembolic risk, especially due to the use of whole blood samples, thus including the contribution of cellular components on hemostasis, when compared to tests using citrated plasma [2]. Meanwhile, the tests carried out in this study have advantages over TEG because it details which elements are involved on the possibility of increased thromboembolic risk, a fact that cannot be studied and detailed by using TEG.

In human, the hypercoagulable condition has been associated to hyperadrenocorticism due to an increase of factor VIII activity, or to associated increased factors VIII, V, and prothrombin, or by increased von Willebrand factor and factors VIII, IX, XI, and XII, and platelet hyperactivity [2]. Many authors have tried to correlate thromboembolism associated to hyperadrenocorticism with increased procoagulant factors and diminished fibrinolytic system in dogs [2,6]. Studies where authors have tried to confirm the correlation between thromboembolism and hypercortisolism have not evaluated the possibility of concurrent morbid processes [32,33], which are very common findings in human beings affected by hyperadrenocorticism, such as insulin resistance and systemic arterial hypertension [2].

The chronic corticotherapy is a well-known risk factor for thrombosis in dogs, since a 7-day prednisone protocol at 1.0 mg/kg/day resulted in thromboelastographic changes in healthy dogs. Although it is impossible to determine if prednisone administration is the only cause, it is reasonable to conclude that glucocorticoids may contribute to a hypercoagulable condition [34]. A study made by Rose et al. proved the hypercoagulable condition using both anti-inflammatory and immunosuppressive doses in dogs treated for two weeks with prednisone, which was confirmed by thromboelastographic changes [10].

The lack of statistical difference in the parameters between anti-inflammatory and immunosuppressive doses, observed by Rose et al. [10] and the present study, suggests that there is no dose-dependent effect of prednisone on the hemostatic system in a two-week protocol.

## Conclusions

It is not possible to infer that prednisone administration does not cause a thromboembolic risk in dogs, since the decrease in antithrombin levels with anti-inflammatory and immunosuppressive doses did not reach clinical significant levels. There is no evidence of prednisone dose-dependent activity on hemostasis. Although thromboembolic risk was not associated to exogenous hypercortisolism in healthy dogs, the occurrence of other systemic conditions should be taken into consideration on corticotherapy.

## Competing interests

The authors declare that they have no competing interests.

## Authors’ contributuons

FGR and RKT designed the study. FGR, EFC, CRSM and RKT performed the laboratorial analysis. FGR, EFC and RKT collected the samples. FGR and RKT performed the data analysis, reviewed the literature and prepared the manuscript. All authors read and approved the final manuscript.
